# A behaviorally related developmental switch in nitrergic modulation of locomotor rhythmogenesis in larval *Xenopus* tadpoles

**DOI:** 10.1152/jn.00283.2015

**Published:** 2016-01-13

**Authors:** Stephen P. Currie, Denis Combes, Nicholas W. Scott, John Simmers, Keith T. Sillar

**Affiliations:** ^1^School of Psychology and Neuroscience, University of St. Andrews, St. Andrews, Fife, Scotland, United Kingdom; and; ^2^Institut de Neurosciences Cognitives et Intégratives d'Aquitaine, Université de Bordeaux, CNRS UMR 5287, Bordeaux, France

**Keywords:** locomotion, tadpole, development, nitric oxide, modulation

## Abstract

Locomotor control requires functional flexibility to support an animal's full behavioral repertoire. This flexibility is partly endowed by neuromodulators, allowing neural networks to generate a range of motor output configurations. In hatchling *Xenopus* tadpoles, before the onset of free-swimming behavior, the gaseous modulator nitric oxide (NO) inhibits locomotor output, shortening swim episodes and decreasing swim cycle frequency. While populations of nitrergic neurons are already present in the tadpole's brain stem at hatching, neurons positive for the NO-synthetic enzyme, NO synthase, subsequently appear in the spinal cord, suggesting additional as yet unidentified roles for NO during larval development. Here, we first describe the expression of locomotor behavior during the animal's change from an early sessile to a later free-swimming lifestyle and then compare the effects of NO throughout tadpole development. We identify a discrete switch in nitrergic modulation from net inhibition to overall excitation, coincident with the transition to free-swimming locomotion. Additionally, we show in isolated brain stem-spinal cord preparations of older larvae that NO's excitatory effects are manifested as an increase in the probability of spontaneous swim episode occurrence, as found previously for the neurotransmitter dopamine, but that these effects are mediated within the brain stem. Moreover, while the effects of NO and dopamine are similar, the two modulators act in parallel rather than NO operating serially by modulating dopaminergic signaling. Finally, NO's activation of neurons in the brain stem also leads to the release of NO in the spinal cord that subsequently contributes to NO's facilitation of swimming.

locomotion, like most other rhythmic motor behaviors, requires considerable flexibility to adapt movement to changing environmental, developmental and organismal demands. This functional plasticity, which resides partly within the underlying central motor networks, occurs over multiple time scales, from rapid adaptive changes in response to fast-acting sensory inputs to persistent operational changes as motor circuitry is assembled and refined to match emerging behavioral requirements during ontogeny.

The basic network controlling locomotor behavior, termed “central pattern generator” (CPG), produces a patterned motor output that drives coordinated muscle contractions suitable for generating body propulsion. CPGs are subject to modulatory influences that can be either intrinsic, that is, derived from CPG network neurons themselves (e.g., Dale and Gilday 1996), or extrinsic, arising from neurons belonging to supraspinal systems (reviewed in Katz 1995; Miles and Sillar 2011). Among the best described extrinsic sources of modulatory input to spinal CPGs are the biogenic amines [serotonin (5-HT); noradrenaline (NA); and dopamine (DA)]. However, the free-radical gaseous signaling molecule nitric oxide (NO) has also been shown to be involved in the modulation of locomotion in several vertebrate species, including larval *Xenopus* frog (McLean and Sillar 2000, 2002, 2004), lamprey (Kyriakatos and El Manira 2007; Kyriakatos et al. 2009; Song et al. 2012) and mice (Foster et al. 2014). In hatchling *Xenopus* tadpoles, prior to the onset of free-swimming behavior (from stages 37/38 to 42; Nieuwkoop and Faber 1956), NO exerts a net inhibitory effect by shortening swim episodes and simultaneously decreasing the frequency of motor bursts within episodes (McLean and Sillar 2000). The cellular and synaptic effects of NO at these early postembryonic tadpole stages involve the simultaneous presynaptic facilitation of both GABAergic and glycinergic fast inhibitory signaling. Thus, although NO also causes a direct depolarization of spinal neurons (McLean and Sillar 2002, 2004), the overall inhibition of swimming suggests that this potentially excitatory effect is overridden by NO's facilitation of inhibitory synaptic transmission. The endogenous effects of NO are presumed to involve nitrergic signaling in the brain stem, where three bilaterally paired clusters of NO synthase (NOS)-positive neurons are located in relatively close proximity to descending modulatory neurons, including GABAergic, noradrenergic and serotonergic populations (McLean and Sillar 2000, 2001, 2004). In early stage (up to 42) *Xenopus*, NO acts as a “metamodulator” by enhancing NA release in the brain stem, which in turn slows evoked swimming by facilitating glycinergic transmission in the spinal cord (McLean and Sillar 2004). In addition, NO and dopaminergic signaling have been found to interact directly in other systems, such as in the striatum where NO enhances the amine's effects via DA re-uptake blockade (Kiss et al. 1999). In *Xenopus* tadpoles, spinal DA receptor activation exerts diverse modulatory influences on swimming occurrence (Clemens et al. 2012), raising the possibility that descending dopaminergic pathways could be intercalated between the brain stem nitrergic system and its influence on downstream spinal CPG circuitry.

While the tadpole's brain stem nitrergic populations are already present at the time of hatching, NOS-positive neurons subsequently appear in the spinal cord from stage 47, suggesting additional as yet unknown roles for NO later in larval development (McLean and Sillar 2001; Ramanathan et al. 2006). Here, to begin to address this issue, we first trace the temporal emergence of free swimming and its neural correlate in posthatching *Xenopus* tadpoles. We then describe the effects of NO at both early sessile and later free-swimming stages. Our data identify a switch in nitrergic modulation from overall inhibition to excitation, coincident with the transition to a free-swimming mode of locomotion. In addition, we show in isolated brain stem-spinal cord preparations that NO's excitatory effects are evidenced by an increase in the probability of spontaneous swim episode occurrence, and that these effects are mediated within the brain stem, but without involving a metamodulatory interaction with DA signaling. Finally, the activation of neurons in the brain stem leads to the release of NO in the spinal cord that in turn contributes to NO's facilitation of swimming.

## METHODS

### 

#### Animals.

Experiments were performed on both prefeeding sessile larval stages (37/38–44) and on free swimming, pre- and pro-metamorphic stages (45–58) of the South African clawed frog, *Xenopus laevis* (Nieuwkoop and Faber 1956). Animals were obtained by human chorionic gonadotrophin hormone-assisted (1,000 U/ml; Sigma) matings of adults selected from an in-house breeding colony. Fertilized ova were collected and reared in enamel trays until the first free-feeding stages, before being transferred to standard glass aquarium tanks. All procedures conformed with the UK Animals (Scientific Procedures) Act 1986 and the European Community Council directive of 24 November 1986 (86/609/EEC) and were approved by the University of St. Andrews Animal Welfare Ethics Committee and the local ethics committee of the University of Bordeaux (no. 3301100012-A).

#### Larval swimming behavior.

Individual tadpoles were placed under HEPES saline (composition in mM: 115 NaCl, 2.5 KCl, 2.5 NaHCO_3_, 10 HEPES, 1 MgCl_2_, 4 CaCl_2_, pH 7.4, with NaOH) in a shallow circular transparent glass dish (5-cm diameter) sitting on top of graph paper. Swimming behavior was recorded for 5 min per animal using a Casio EXILIM digital camera (image acquisition frequency, 480 frames/s). Displacement (in cm) was calculated by manually summating the total displacement from each frame of a recorded sequence. Similar frame-by-frame observations allowed determining the time that an animal spent spontaneously swimming as a percentage of overall time.

#### In vitro preparations and electrophysiology.

Stage 37/38–44 posthatching larvae were immobilized in α-bungarotoxin, as previously described (e.g., McLean and Sillar 2000). For later stages, prior to electrophysiological experiments, the animals were deeply anesthetized according to standard Schedule 1 methods (UK Home Office) by exposure to ∼230 μg/ml ethyl 3-aminobenzoate methanesulfonate (MS222); then the heart and forebrain rostral to its junction with the midbrain were removed.

For semi-intact preparations of stages 45–47, animals subjected to Schedule 1 procedure were immobilized in α-bungarotoxin and placed in a recording chamber with circulating HEPES saline. The skin over the trunk and tail myotomes was then removed, and suction electrodes were placed in the intermyotomal clefts to record spinal ventral root activity.

For pro-metamorphic stages 48–62, animals were prepared and dissected as described previously (e.g., Combes et al. 2004). In addition, an electrical stimulating electrode was sometimes placed against the optic tectum to evoke episodes of fictive locomotion by short trains (1 to 5 pulses, depending on the preparation) of 1-ms pulses up to 50 V delivered via a DS2A isolated stimulator (Digitimer).

#### Pharmacological manipulations.

Drugs used during electrophysiological experiments were as follows: NO donors *S*-nitroso-*N*-acetyl penicillamine (SNAP, 200 μM) and diethylamine NONOate (DEA-NO, 50–200 μM); the NO scavenger 2-phenyl-4,4,5,5-tetramethylimidazoline-1-oxyl 3-oxide (PTIO, 50–200 μM); the NOS inhibitor *N*^ω^-nitro-l-arginine methyl ester hydrochloride (l-NAME, 1–2 mM) and the D1-like DA receptor antagonist R(+)-SCH-23390 hydrochloride (SCH; 50 nM). Drugs were dissolved in distilled H_2_O (18 MΩ), except for PTIO, which was dissolved in ethanol (0.1%); as previously described (Reith and Sillar 1997), the vehicle alone was found to have no observable effect on the preparations. All drugs were initially bath-applied through an open-loop peristaltic pump supply that was switched to closed-loop circulation after 15 min. By this time, the volume of the bath had been exchanged approximately three to five times. Recordings were made throughout the ensuing 30- to 60-min period of drug exposure. Drugs were washed out using control saline, and, for experiments requiring multiple drug exposures, at least 30 min of washout were performed prior to subsequent drug applications. For a series of so-called “split-bath” experiments, a transverse Vaseline wall was constructed by syringe ejection at the level of the brain stem/spinal cord junction to enable selective drug applications to either of these two central nervous system (CNS) regions.

#### Data acquisition and statistical analysis.

Extracellular signals were amplified using differential AC amplifiers (A-M Systems model 1700), digitized with a CED1401 interface (Cambridge Electronic Design, Cambridge, UK) and processed and stored on a PC computer using Spike 2 (CED) software. Electrophysiological data were analyzed using Dataview software (version 8.62, courtesy of W. J. Heitler, School of Biology, University of St. Andrews, St. Andrews, UK). Statistical analyses were performed in SPSS (version 21; IBM). The displacement and time spent active (see above) for the different animal stages were compared with these values in stage 37/38 using either an independent samples *t*-test or a Mann-Whitney *U*-test for nonparametric data. For comparison of drug effects, repeated-measures ANOVAs with Bonferroni post hoc corrections were used. For comparisons between experiments, values were normalized to the corresponding control. In these cases, data were analyzed using a related-samples Wilcoxon signed rank test. Error bars in all figures represent SE of the mean.

## RESULTS

### 

#### Postembryonic development of free swimming.

**Fig. 1.**
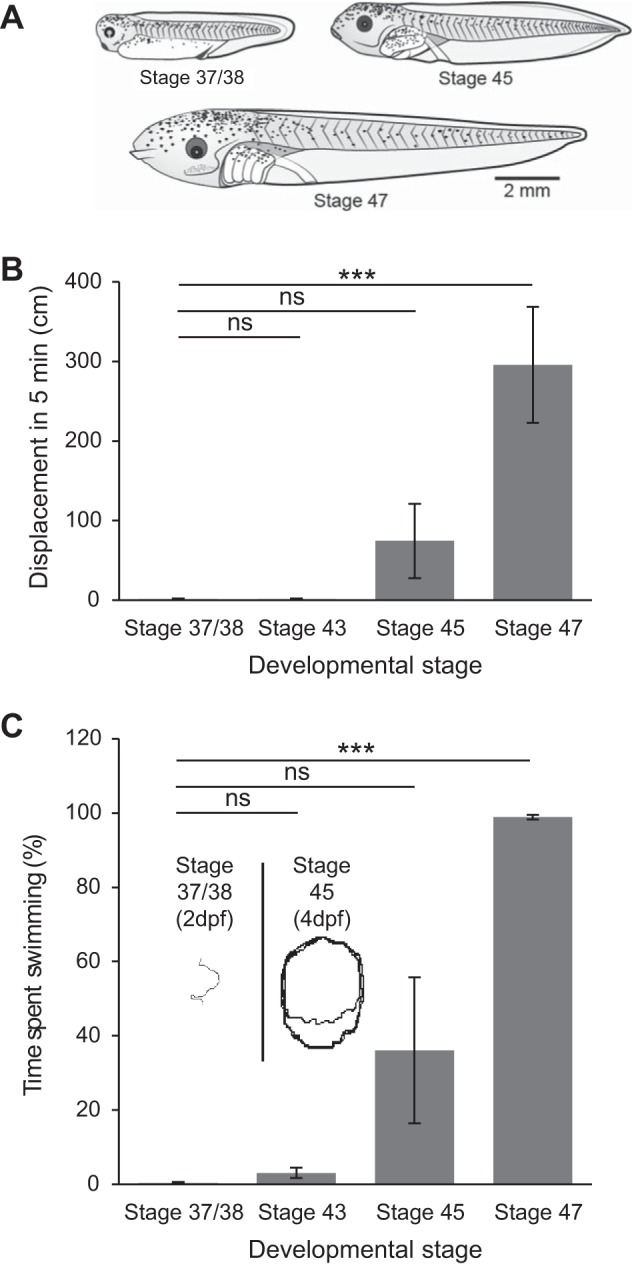
Developmental increase in spontaneous swimming behavior. *A*: examples of postembryonic stage 37/38 and larval stages 45 and 47 *Xenopus laevis* tadpoles. Drawings kindly provided by Laurence D. Picton, with permission. *B*: bar graph of the displacement during swimming measured over a 5-min period at different larval stages. *C*: bar graph of percentage of total time spent swimming measured over 5 min at different larval stages. *Insets* show typical swim trajectories at stages 37/38 [2 days postfertilization (dpf)] and 45 (4 dpf). Values are means ± SE. ns, Nonsignificant. ***P < 0.001.

Newly hatched *Xenopus laevis* tadpoles [Fig. 1; stage 37/38, ∼2 days postfertilization (dpf)] display very little spontaneous locomotion and, unless stimulated, lead a mainly dormant life until they progressively adopt a more motile existence at the onset of active feeding (stage 45, ∼4 dpf; Muntz 1975; van Mier et al. 1989). The motor behavior of tadpoles was measured from stage 37/38 through to 47 when the limb buds are just starting to form (Nieuwkoop and Faber 1956). A gradual increase in spontaneous swimming activity was observed from stage 45, as evidenced by the displacement of animals measured over a 5-min period (Fig. 1*B*). At stage 37/38, animals swam on average 1.38 ± 0.63 cm in 5 min (*n* = 4); stage 43, 1.50 ± 0.81 cm (*n* = 6); stage 45, 74.50 ± 46.80 cm (*n* = 6) and stage 47 animals swam 295.8 ± 72.73 cm (*n* = 5). Stage 47 animals thus swam significantly further (*P* = < 0.01) than at all earlier stages in development, suggesting free swimming has fully emerged at this point in development. A similar result was obtained when the time spent active during 5 min was measured (Fig. 1*C*): stage 37/38 animals swam on average 0.42 ± 0.21% of the time recorded; stage 43, 3.11 ± 0.16%; stage 45, 36.06 ± 19.64%; and stage 47 animals swam 98.87 ± 0.61% of the time. Again stage 47 animals were significantly more active than all earlier stages (*P* < 0.01). These experiments revealed not only that stage 47 tadpoles spontaneously swam significantly further than their stage 37/38 counterparts, but also that the nature of the swimming they expressed was different. Stage 37/38 tadpoles swam in a much more episodic fashion and so rarely that each episode had a clear beginning and end. In contrast, stages 45 and 47 swam around the dish almost constantly and displayed a greater progressive waxing and waning of propulsion compared with the all-or-none expression of swimming observed in younger prefeeding animals (see *insets* in Fig. 1*C* showing example swim trajectories for stages 37/38 and 45). Significantly, this switch in locomotor behavior to an increasingly free-swimming lifestyle coincides precisely with the period when tadpoles have consumed their yolk sac and commenced their free-feeding larval existence. Presumably, this developmental change in behavior reflects underlying maturational changes in spinal locomotor circuit operation and/or its control by descending modulatory influences from the brain stem.

#### Increase in spontaneous fictive swimming with development.

**Fig. 2.**
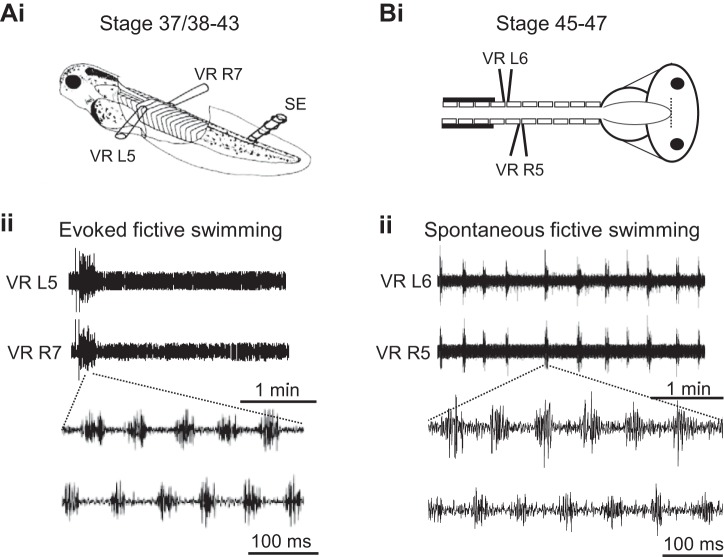
Comparison of fictive swimming in *Xenopus* tadpoles at stages before and after onset of free feeding. *Ai*: preparation used at stages 37/38–44; schematic shows a stage 42 animal immobilized in α-bungarotoxin with locations of extracellular ventral root (VR) recording electrodes on left (L) and right (R) sides and a tail-skin stimulating electrode (SE). *Aii*: a single episode of fictive swimming evoked by a 1-ms electrical current pulse applied via the SE. Note that, after the end of the swim bout, the preparation remains silent in the absence of further stimulation. Below is an expanded excerpt during swimming activity highlighting L/R alternation of VR bursts. *Bi*: equivalent preparation at stage 45–47. *Bii*: in the absence of any extrinsic stimulation, the preparation spontaneously generates regular episodes of fictive swimming, here, every ∼20 s. Below is an expanded excerpt of swimming activity during the indicated episode again showing L/R VR burst alternation.

**Fig. 3.**
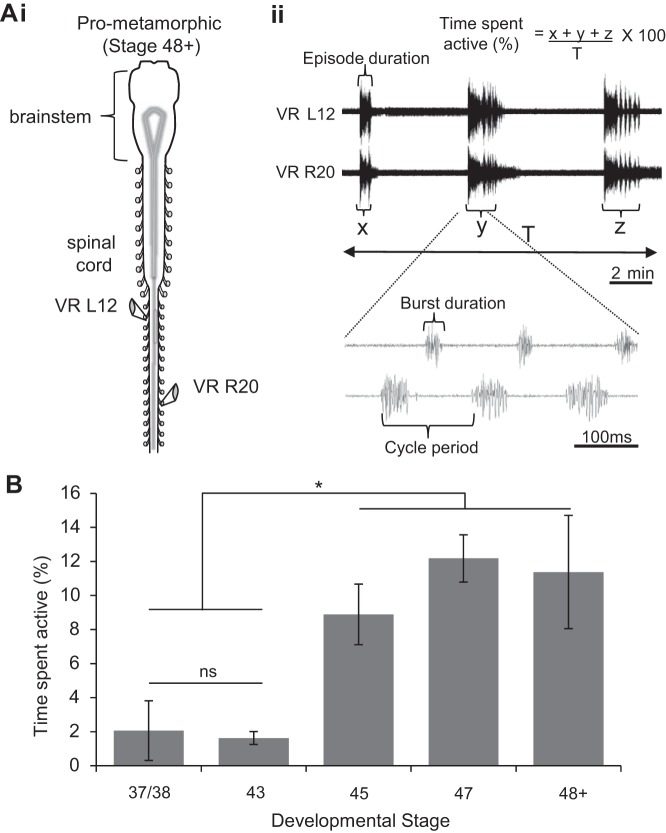
Development of fictive swimming in later stage *Xenopus* tadpoles. *Ai*: completely isolated central nervous system (CNS) preparation at pro-metamorphic (48–62) larval stages with recording electrodes (at VR L12, R20). *Aii*: repetitive bouts of spontaneous fictive swimming. Shown below is an expanded excerpt of fictive swimming and method used to calculate the percentage of time spent active (see also at *top*). Other measured rhythm parameters within swim episodes, burst duration (BD), cycle period (CP) and episode duration (ED), are also indicated. *B*: bar graph illustrating the developmental increase in spontaneous fictive swim occurrence expressed as a mean percentage of time (over a 5-min period) that preparations were active. Values are means ± SE. *P < 0.05.

Fictive swim bouts in immobilized tadpoles rarely occur spontaneously between stages 38/37 and 42 but can be reliably evoked by sensory stimulation via a current pulse applied to the tail skin (Fig. 2*Ai*). Such episodes of fictive swimming have defined onsets and terminations and consist of left-right alternating bursts of activity in spinal ventral roots innervating the tail muscles (Fig. 2*Aii*). To further explore the development of *Xenopus* locomotion, a semi-intact preparation (see methods) was used to record fictive swimming from older larvae (Fig. 2*Bi*), and stages 45 to 47 in particular, since this range corresponds to the onset of free-swimming, as shown by our behavioral data. In contrast to younger larvae (Fig. 2*Bi*), the locomotor CPG activity displayed by such stage 45–47 preparations consisted of regularly repeating fictive swim bouts that occurred spontaneously (Fig. 2*Bii*), consistent with the emergence of an inherent capability to initiate locomotor activity, as indicated by our behavioral data (see Fig. 1, *B* and *C*). To further investigate this maturational change in locomotor expression, we used completely isolated brain stem-spinal cord preparations of even later stage 48–62 tadpoles (Combes et al. 2004), thereby covering the entire pre- and pro-metamorphic larval period. Such in vitro CNS preparations (Fig. 3*Ai*) also continued to generate spontaneous bouts of rhythmic ventral root bursting (Fig. 3*Aii*), appropriate for driving undulatory axial swimming movements in the intact animal. Figure 3*B* shows the mean durations (expressed as a percentage of overall recording period) of spontaneous fictive swimming expressed by the various in vitro preparations from the different larval stages; there was a significant increase at stage 45 (8 ± 1.78%; *n* = 6, *P* < 0.05) and stage 47 (12.18 ± 1.39%; *n* = 5, *P* < 0.05) compared with stages 37/38–42 (2.06 ± 1.75%, *n* = 6) and stage 43 (1.63 ± 0.38%; *n* = 5, nonsignificant), which themselves were not significantly different. In addition, pro-metamorphic stages were also more spontaneously active (11.38 ± 3.33%; *n* = 8, *P* < 0.05) than stage 37/38 and stage 43 animals. Thus together these results obtained from semi-intact through to completely isolated CNS preparations show an equivalent developmental profile to that of the behavioral studies reported in Fig. 1*C*. This in turn indicates that the increase in spontaneous swimming activity in animals from the onset of free-feeding derives largely from changes within the CNS motor circuitry itself.

#### Modulation of fictive swimming by NO.

**Fig. 4.**
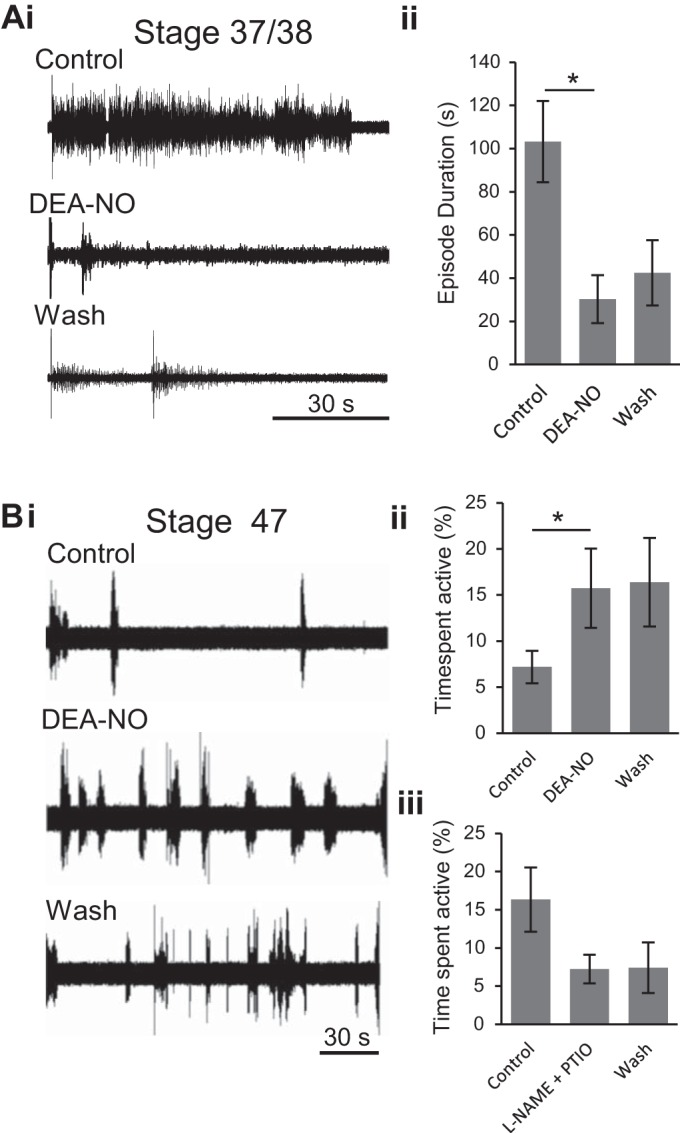
Developmental switch in nitric oxide (NO) modulation of fictive swimming in *Xenopus* tadpoles. *Ai*: extracellular recording (VR R5) from a stage 42 tadpole (as in Fig. 2*Ai*) of evoked episodes of fictive swimming in control, in the presence of the NO donor diethylamine NONOate (DEA-NO; 200 μM) and during drug washout. *Aii*: pooled data depicting the mean ED in each condition (*n* = 9). *Bi*: extracellular record (VR R7) from a stage 47 tadpole (as in Fig. 2*Bi*) of spontaneous fictive swimming occurring in control conditions and when it increased in the presence of DEA-NO (200 μM). *Bii*: pooled data showing mean percentage of time spent active in each condition (*n* = 5). *Biii*: pooled data showing the mean time spent active following co-application of the NOS inhibitor *N*^ω^-nitro-l-arginine methyl ester (l-NAME; 1 mM) and the NO scavenger 2-phenyl-4,4,5,5-tetramethylimidazoline-1-oxyl 3-oxide (PTIO; 100 μM, *n* = 4). *P < 0.05.

The inhibitory effects of the NO donor SNAP on fictive swimming between stages 37/38 and 42 have been reported previously (McLean and Sillar 2000, 2004). To confirm and extend these data using a different and reportedly more potent NO donor (Ferrero et al. 1999), we applied DEA-NO (200 μM) to stage 37/38 and 42 preparations (Fig. 4*A*). In both cases, similar results to those already documented were observed, in that the durations of swim episodes were dramatically reduced (Fig. 4, *Ai* and *Aii*). In nine preparations, the presence of DEA-NO significantly reduced evoked episode durations (EDs) from a mean value of 103.2 ± 18.8 s to 30.3 ± 11.1 s (*P* < 0.05), which did not wash, increasing to just 42.5 ± 15.1 s (Fig. 4*Aii*). Moreover, the presence of either donor did not affect the actual occurrence of swim episodes, which should be remembered almost never occur spontaneously and require sensory triggering (by tail skin stimulation) at these early larval stages.

In direct contrast to these earlier larval stages, DEA-NO (200 μM) increased swimming activity in stage 47 tadpoles (Fig. 4*Bi*), as indicated by a significant increase in the mean percentage of time spent active from 7.18 ± 1.74% to 15.73 ± 4.31% (*n* = 5, *P* < 0.05; Fig. 4*Bii*). Alternatively, reducing the presence of any endogenous NO in a different set of preparations at this stage, by bath application of the NOS inhibitor l-NAME and the NO scavenger PTIO, reduced the mean time spent active from 16.34 ± 4.21% in control to 7.24 ± 1.90% following drug application (*n* = 4; Fig. 4*Biii*). This apparent decrease was not statistically significant (*P* = 0.136), however, indicating that endogenously produced NO is unable to consistently activate the swimming rhythm at this stage in development (but see below and discussion). Thus, while NO has a net inhibitory effect on evoked fictive swimming at earlier postembryonic stages, by the first free-swimming stages, exogenous NO, at least, exerts the opposite effect by increasing spontaneous locomotor output in these older stage 47 preparations.

**Fig. 5.**
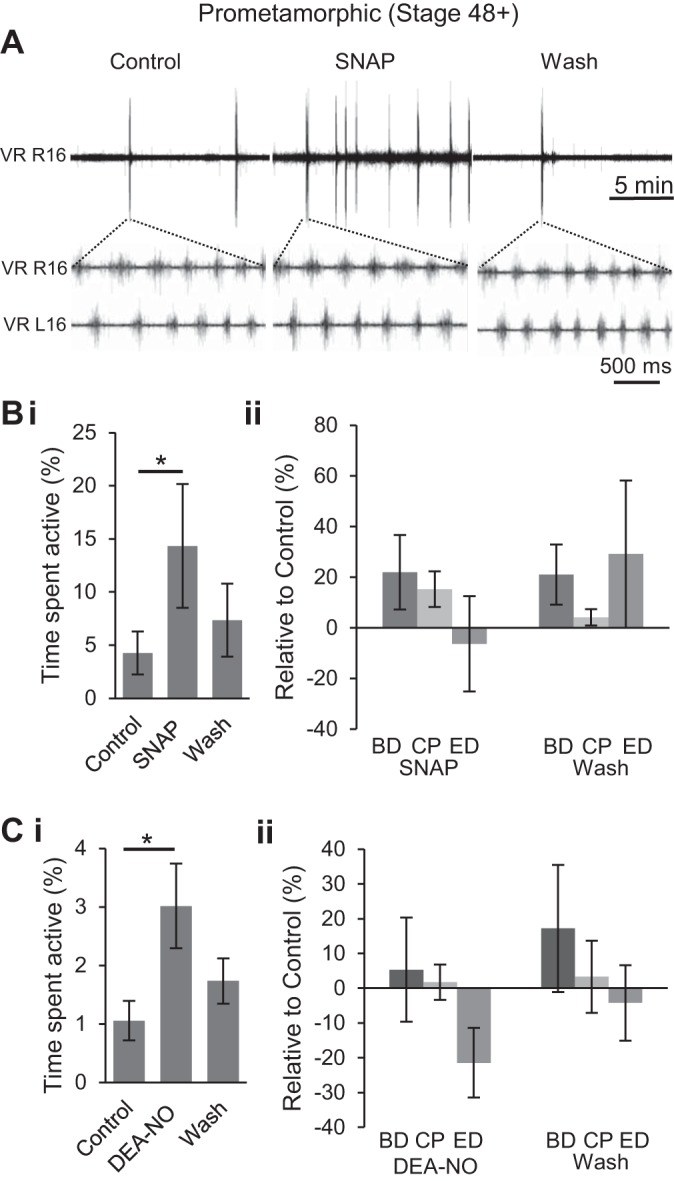
NO donors increase the occurrence of spontaneous locomotor activity at pro-metamorphic stages. *A*: VR recording from a stage 54 tadpole both prior to, and during, NO donor [*S*-nitroso-*N*-acetyl penicillamine (SNAP), 200 μM] application and then following washout with expanded excerpts of L/R alternating VR (L/R 16) bursting during fictive swimming in each condition. *Bi*: pooled data showing the mean percent time spent active in each condition (*n* = 13). *Bii*: pooled data from the same preparations [as illustrated in expanded excerpts in *A* showing mean BD, CP, and ED (see Fig. 3*Aii*)] relative to control values during and after SNAP application. *C*: same pooled data analyses as in *B*, but for application of a different NO donor, DEA-NO (50–200 μM; *n* = 14). Values are means ± SE. *P < 0.05.

To further investigate the effects of NO and establish whether this apparent switch in NO's role persists into later developmental stages or instead represents a transient, stage-specific modification, we tested nitrergic manipulation on isolated brain stem-spinal cord preparations from older pre- and pro-metamorphic (stage 48–62) animals. At all stages in this later period and irrespective of the NO donor applied (i.e., SNAP, Fig. 5, *A* and *B*; DEA-NO, Fig. 5*C*), NO consistently increased the total duration of swimming activity, as observed in younger stage 47 preparations. The addition of SNAP, for example, increased the total time spent active by threefold from a mean of 4.25 ± 2.02% in control to 14.33 ± 5.82% (*n* = 13, *P* < 0.05; Fig. 5, *A* and *Bi*). Upon return to control saline, total activity levels returned to control mean values (7.34 ± 3.42%; Fig. 5, *A* and *Bi*). However, other parameters of the swimming pattern, including burst duration (BD), cycle period (CP) and ED, were not significantly affected by this NO donor (Fig. 5*Bii* and see Table 1). Similarly, bath application of DEA-NO (50–200 μM) caused an increase in the total duration of fictive swimming from a mean of 1.06 ± 0.34% in control to 3.02 ± 0.72% (*n* = 14, *P* < 0.05; Fig. 5*Ci*). Upon washout, activity returned toward control levels (1.73 ± 0.39%; Fig. 5*Ci*). Again, the other fictive swim parameters (BD, CP, ED) were not significantly affected (Fig. 5*Cii* and see Table 1). Taken together, these results indicate that exogenous NO increases the occurrence of spontaneous locomotor activity but without significantly affecting any other temporal parameters of the swimming rhythm. Thus, the switch in NO's effect on locomotor behavior from an overall inhibitory to excitatory influence that takes place by stage 47 persists throughout remaining larval development.

**Table 1.** Nitrergic drugs on pro-metamorphic stages (stage 48+)

	SNAP			DEA-NO			l-NAME			PTIO		
Tissue Exposed/Parameter	Control	Drug	Wash	Control	Drug	Wash	Control	Drug	Wash	Control	Drug	Wash
Whole preparation												
Swim, %	4.25 ± 2.03	14.33 ± 5.82	7.34 ± 3.42	1.06 ± 0.34	3.01 ± 0.72	1.73 ± 0.39	10.42 ± 1.60	4.33 ± 0.88	7.91 ± 1.52	4.62 ± 1.60	1.37 ± 0.37	3.52 ± 10.65
Burst duration, ms	82.40 ± 7.66	89.39 ± 8.87	89.07 ± 7.93	128.20 ± 33.99	107.50 ± 21.29	109.81 ± 19.93	70.35 ± 7.69	79.54 ± 9.19	73.00 ± 5.16	70.90 ± 7.13	91.02 ± 18.85	82.38 ± 12.29
Cycle period, ms	195.68 ± 11.18	223.92 ± 15.97	200.56 ± 13.19	211.78 ± 16.24	213.27 ± 11.02	217.45 ± 17.88	196.57 ± 7.58	204.31 ± 12.47	198.56 ± 11.66	197.88 ± 15.10	207.19 ± 17.97	206.01 ± 14.87
Episode duration, s	4.25 ± 2.03	14.33 ± 5.82	7.34 ± 3.42	1.06 ± 0.34	3.01 ± 0.72	1.73 ± 0.39	10.42 ± 1.60	4.33 ± 0.88	7.91 ± 1.52	5.23 ± 1.15	5.03 ± 1.28	3.66 ± 0.61

	SNAP and DEA-NO			l-NAME and PTIO		
	Control	Drug	Wash	Control	Drug	Wash
Spinal cord						
Swim, %	2.92 ± 2.02	2.46 ± 1.83	1.02 ± 0.49	1.15 ± 0.31	0.86 ± 0.14	0.90 ± 0.18
Burst duration, ms	89.42 ± 12.09	96.17 ± 18.50	85.00 ± 7.67	90.64 ± 12.77	107.01 ± 19.95	90.70 ± 13.61
Cycle period, ms	252.35 ± 13.67	268.73 ± 32.86	239.38 ± 18.00	206.64 ± 18.25	220.85 ± 21.37	186.63 ± 17.24
Episode duration, s	3.46 ± 0.98	2.38 ± 0.44	2.55 ± 0.49	2.49 ± 0.79	2.56 ± 0.89	3.40 ± 1.10
Brain stem						
Swim, %	1.04 ± 0.48	1.61 ± 0.44	1.01 ± 0.40	3.97 ± 3.14	0.44 ± 0.20	3.59 ± 2.86
Burst duration, ms	85.00 ± 7.67	98.26 ± 12.68	81.60 ± 6.08	93.39 ± 11.81	110.98 ± 18.73	83.99 ± 8.68
Cycle period, ms	239.38 ± 18.00	251.09 ± 24.12	220.50 ± 11.86	168.96 ± 11.90	178.18 ± 17.90	180.46 ± 7.87
Episode duration, s	2.49 ± 0.49	2.22 ± 0.28	3.11 ± 0.85	2.59 ± 0.90	2.44 ± 1.53	2.57 ± 0.77

Values are means ± SE. SNAP, *S*-nitroso-*N*-acetyl penicillamine; DEA-NO, diethylamine NONOate; l-NAME, *N*^ω^-nitro-l-arginine methyl ester; PTIO, 2-phenyl-4,4,5,5-tetramethylimidazoline-1-oxyl 3-oxide.

#### Inhibition of fictive swimming by NO removal.

**Fig. 6.**
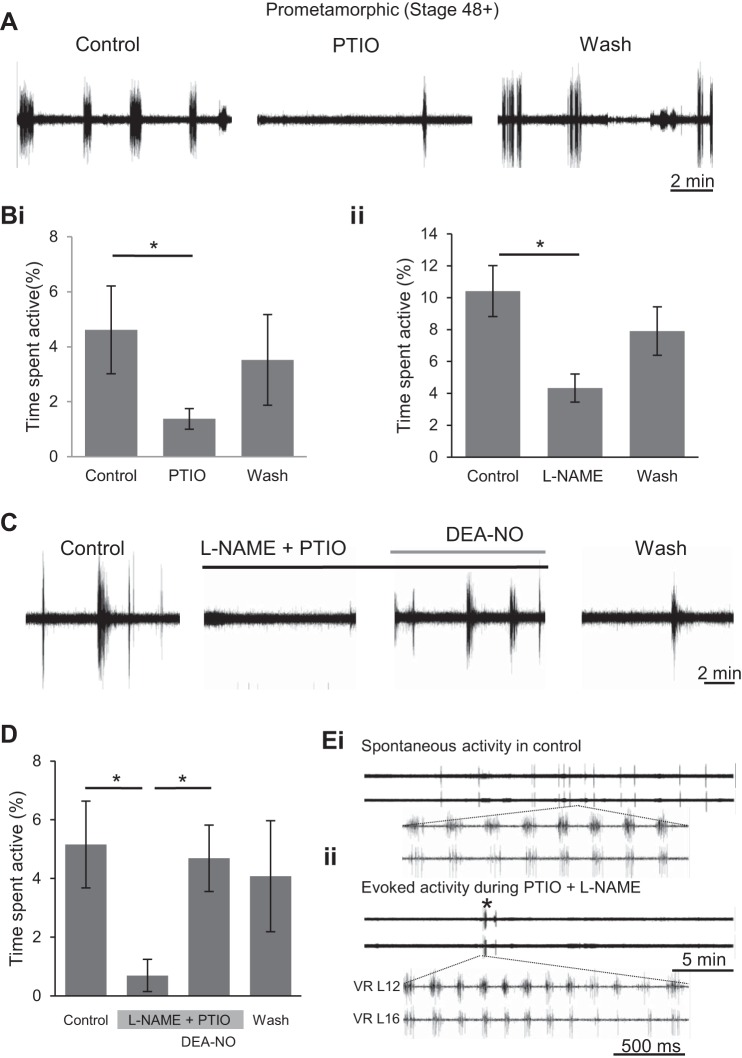
Scavenging endogenous NO and inhibiting endogenous NO synthase (NOS) activity decreases fictive locomotion occurrence. *A*: VR recordings (VR L17) from a stage 55 tadpole before, during bath application and following washout of the NO scavenger PTIO (50–200 μM). *Bi*: pooled data of the mean time spent active in control, under PTIO application and during washout (*n* = 10). *Bii*: pooled data of the mean percentage of time spent active before, during and after application of the NOS inhibitor l-NAME (1–2 mM; *n* = 6). *C*: VR recordings (VR R18) from a stage 55 tadpole of locomotor activity following sequential applications of combined PTIO (100 μM) and l-NAME (1 mM), and then NO donor DEA-NO (200 μM). *D*: pooled data of the mean percentage of time spent active during the drug manipulations in *C* (*n* = 4). Values are means ± SE. *P < 0.05. *E*: during prolonged exposure to PTIO and l-NAME, spontaneous locomotor activity was generally completely abolished (c.f., *top* traces in *Ei* and *Eii*; also see *C* and *D*). However, the central pattern generator (CPG) network remained functional during this drug-induced silence since electrical stimulation of the optic tectum (* in *Eii*) could elicit episodes of rhythmic activity that were very similar from spontaneous fictive locomotion in control (c.f., *bottom* traces in *Ei* and *Eii*).

Scavenging endogenous NO with PTIO (50–200 μM) in older larval stages (stage 48+) had the opposite effect to SNAP or DEA-NO in that PTIO's presence resulted in a decrease in fictive swimming activity from 4.62 ± 1.60% in control to 1.37 ± 0.37%. Following washout, activity returned toward control levels to 3.52 ± 1.65% (*n* = 10, *P* < 0.05; Fig. 6, *A* and *Bi*). Scavenging endogenous NO also had no significant effect on the other parameters of rhythmic bursting within swim episodes (Table 1). The contribution of endogenous NO was further attested to in a different set of experiments by blocking activity of NOS, the enzyme that produces NO, with l-NAME (1–2 mM). Bath application of the enzyme inhibitor significantly decreased time spent active from 10.42 ± 1.60% of total time in control to 4.33 ± 0.88% (*n* = 6, *P* < 0.05; Fig. 6*Bii*). During washout, activity increased back toward control levels to 7.91 ± 1.52%. As for other nitrergic drugs, l-NAME did not consistently alter any other parameter of the locomotor rhythm at these free-swimming larval stages (Table 1).

Although these l-NAME data suggest that NOS-derived NO was responsible for activating swimming, the NO could potentially have arisen from other sources, such as nitroso-thiol intracellular stores (Jaffrey et al. 2001). Furthermore, it is possible that PTIO, which exerted a similar effect to l-NAME (c.f., Fig. 6, *Bi* and *Bii*), was failing to scavenge all available NO from the tissue. Therefore, in a further set of experiments similar to those at stage 47 (Fig. 4*Biii*), l-NAME and PTIO were co-applied (Fig. 6*C*) in an attempt to more completely eliminate endogenous NO from the preparation tissue. Effects similar to those obtained with either drug alone were observed in that fictive swim bout occurrences were substantially reduced or even completely abolished. Specifically, co-applied l-NAME (1 mM) and PTIO (100 μM) together caused a significant reduction in time spent swimming from 5.16 ± 1.48% in control to 0.69 ± 0.55% under drug exposure (*n* = 4, *P* < 0.05; Fig. 6, *C* and *D*).

Although this reduction in spontaneous motor activity appeared to be a specific consequence of the suppressive effect of the drugs on NO signaling, it is also possible that impairing endogenous nitrergic signaling was having a more general, deleterious toxic effect on CNS function that, in turn, was causing swimming activity to decline. However, two lines of evidence argue against the latter possibility. First, subsequent application of the NO donor DEA-NO was able to rescue spontaneous activity expression even in the presence of l-NAME and PTIO, causing a recovery back to control mean levels (*n* = 4, *P* < 0.05; 4.69 ± 1.13%; Fig. 6, *C* and *D*). Moreover, upon washout of all three drugs, activity levels remained similar to control (4.08 ± 1.89%; Fig. 6, *C* and *D*). Second, in preparations which fell completely silent following the removal of endogenous NO by l-NAME and PTIO co-application, the viability of preparations was revealed by evoking swimming activity via electrical stimulation of the optic tectum (see methods; Fig. 6*E*). Taken together, therefore, these findings suggest that the pharmacological reduction of endogenous NO did not compromise locomotor network function per se, but merely reduced its likelihood of being spontaneously active.

#### Site of NO action.

**Fig. 7.**
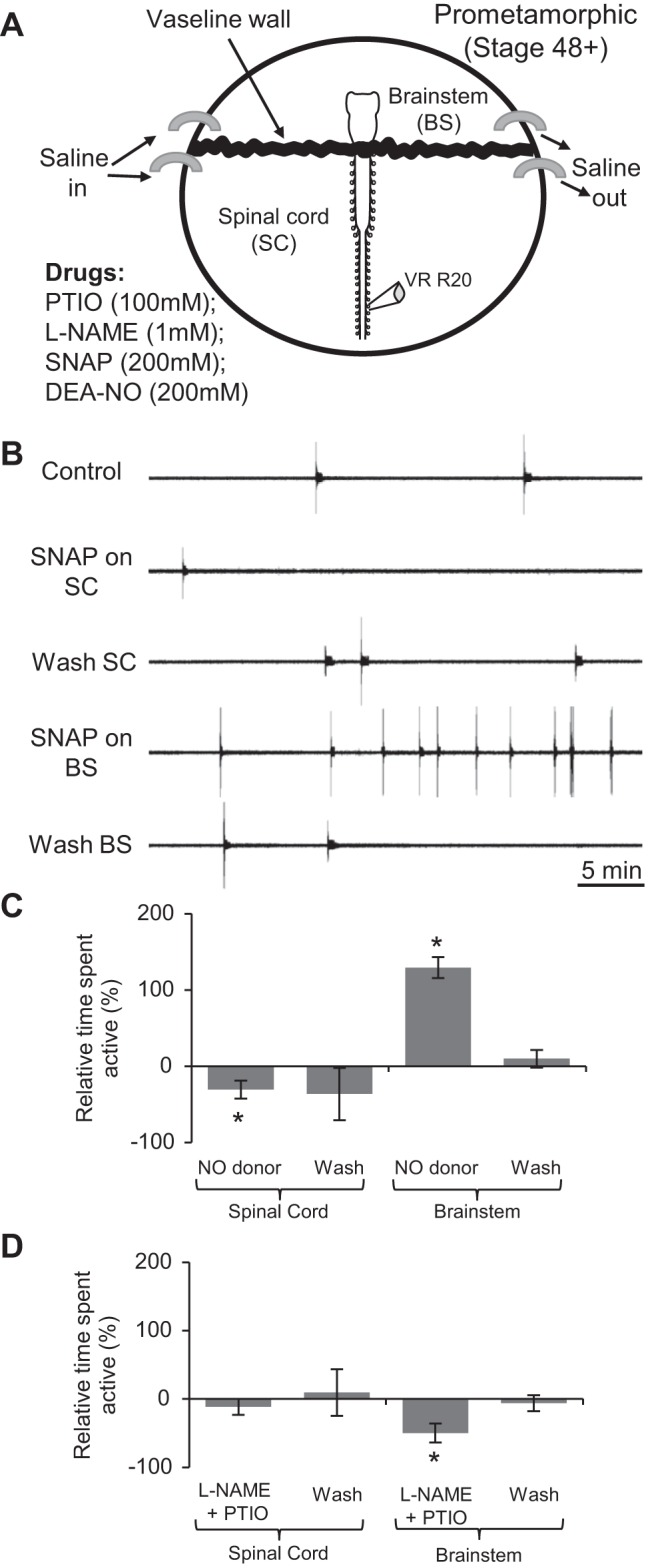
NO's effects on fictive locomotor occurrence at pro-metamorphic stages are mediated in the brain stem (BS). *A*: schematic of the recording set up for split-bath experiments (adapted from Clemens et al. 2012). *B*: VR (VR R20) recording from a stage 54 tadpole showing spontaneous locomotor activity expression during CNS region-specific application of the NO donor SNAP (200 μM). *C*: pooled data for the mean duration of locomotor activity relative to control (0%) during and after application of NO donors SNAP (200 μM) or DEA-NO (50–200 μM) to the spinal cord (SC) alone (*left* bar graphs) or the BS alone (*right* bar graphs) (*n* = 8). *D*: same pooled analyses as in *C*, but for the location-specific co-application of l-NAME and PTIO to either the SC (*left*; *n* = 6) or the BS (*right*; *n* = 5). Values are means ± SE. *P < 0.05.

At earlier postembryonic stages of tadpole development, NO's net inhibition of swimming is proposed to be mediated by modulatory actions occurring in both the brain stem and spinal cord (McLean et al. 2001, 2002, 2004). Our new data show a developmental switch in NO's function, such that, at free-swimming stages, NO increases locomotor episode occurrence, but without other significant effects on swimming performance. We, therefore, asked where along the rostro-caudal neuraxis does this excitatory NO influence take place? In the in vitro preparations of older free-swimming tadpoles, their larger CNS allows the possibility to selectively apply drugs to either the spinal cord or brain stem by using a split-bath recording configuration, and thereby to test the effects of pharmacological manipulations in a more region-specific manner (Fig. 7*A*).

When applied to the spinal cord alone, the NO donors DEA-NO (200 μM; *n* = 4) or SNAP (200 μM; *n* = 4) actually reduced the time spent active to 69.32 ± 11.84% of that in control (*n* = 8, *P* < 0.05; Fig. 7, *B* and *C*). This somewhat surprising effect did not wash fully, although the time spent active following the washout period was overall not significantly different from the value in control (*n* = 8, Fig. 7, *B* and *C*). In contrast, the same drugs applied to the brain stem mimicked the effects of NO donors on the whole preparation (c.f., Fig. 5) by significantly increasing the relative duration of preparation activity to 229.27 ± 58.93% of control (*n* = 8, 5 in DEA-NO and 3 in SNAP, *P* < 0.05; Fig. 7, *B* and *C*). During washout of the drugs from the brain stem, activity durations declined toward control levels, to 109.27 ± 17.10% of control (Fig. 7*C*). During drug applications to either compartment, BD, CP and ED were again not significantly altered (Table 1). These findings, therefore, suggest that the excitatory effects of NO on the expression of locomotor CPG activity are mediated primarily in the brain stem and are able to override any residual inhibitory effects of NO acting in the spinal cord.

In a complementary set of split-bath experiments, application of l-NAME (1 mM) and PTIO (100 μM) to the spinal cord alone did not significantly alter the occurrence of spontaneous locomotor activity relative to control (*n* = 6, Fig. 7*D*). The same drugs applied to the brain stem, however, caused a significant reduction in locomotor output with the time spent active reduced to 50.18 ± 13.78% of control (*n* = 5, *P* < 0.05; Fig. 7*D*). Following drug washout from the brain stem, locomotor output returned to 93.89 ± 11.62 of control levels (Fig. 7*D*). Again, the presence of the drugs in either compartment did not significantly alter the intrinsic swimming rhythm parameters (BD, CP and ED; Table 1). These regional differences in the effects of inhibiting NOS or removing NO on spontaneous fictive swimming thus suggest that a basal release of endogenous NO that facilitates swimming occurs in the brain stem but not in the spinal cord.

#### Direct vs. indirect NO actions.

Interestingly, the excitatory effects of NO described above are qualitatively very similar to those previously reported for D1-like DA receptor activation within the spinal cord at the same developmental stages, in that the amine also increases the probability of swimming occurrence without altering the intrinsic parameters of the swimming rhythm itself (Clemens et al. 2012). We, therefore, tested whether blocking the excitatory effects of DA in pro-metamorphic preparations could diminish NO's ability to influence fictive swimming expression.

**Fig. 8.**
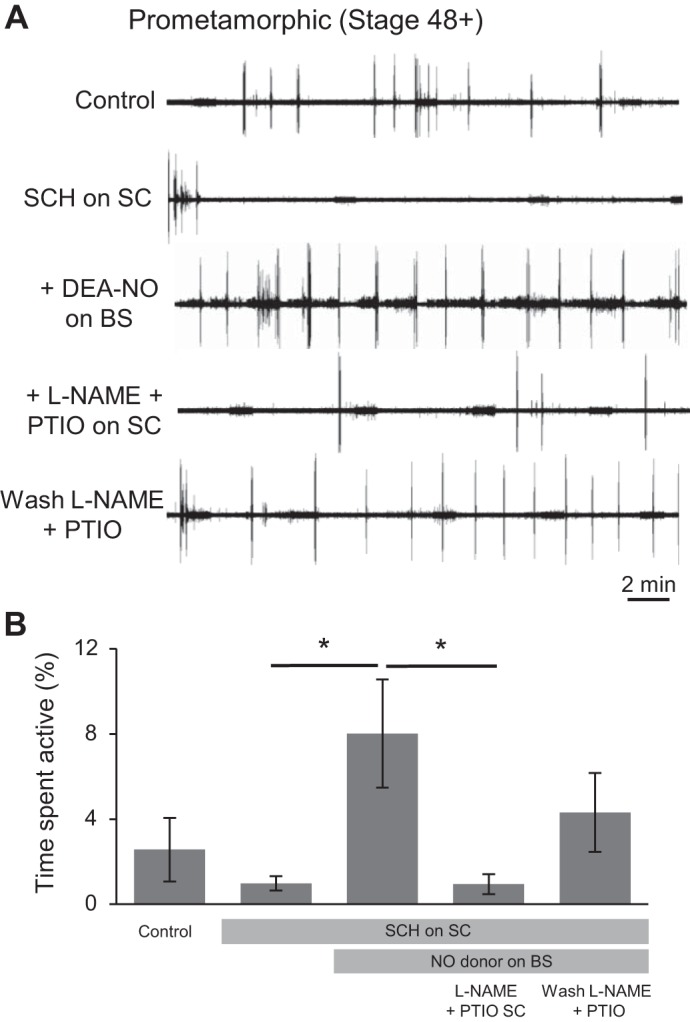
NO's activation of the locomotor CPG via the BS is independent of spinal DA1 receptors, but requires endogenous spinal NO. *A*: VR recording (VR R18) from a stage 55 tadpole showing spontaneous locomotor activity expression during CNS region-specific application of the DA1 receptor antagonist SCH-23390 (SCH; 50 nM), DEA-NO (200 μM), PTIO (100 μM) and l-NAME (1 mM). NB: the regular low-level activity evident in these traces is nonphysiological noise from the Peltier cooling system. *B*: pooled data of the time spent active during the successive drug applications (*n* = 6). Values are means ± SE. *P < 0.05.

In a first step, we suppressed the excitatory effects of DA by blocking excitatory D1 receptors in the spinal cord with the D1-like receptor antagonist SCH (50 nM). We then bath-applied the NO donor DEA-NO (200 μM) to the brain stem compartment. This resulted in a clear and significant increase in the time spent active, from 0.98 ± 0.34% to 8.02 ± 2.54% (*n* = 6, *P* < 0.05; Fig. 8, *A*, third trace, and *B*), comparable with the NO donor effects in the absence of spinal D1 blockade (c.f., Fig. 7*C*). Thus blocking the excitatory D1 pathway failed to occlude the excitatory effects of NO donors. We, therefore, conclude that NO and DA exert similar influences on locomotor network function via parallel rather than serial neuronal pathways.

In a final experimental manipulation, we obtained data suggesting that, following brain stem activation of locomotor output by NO donors, there is a resultant release of NO in the spinal cord (Fig. 8). Indeed, removal of NO specifically from the spinal compartment [using a combination of l-NAME (1 mM) and PTIO (100 μM)] of these preparations, led to a dramatic, repeatable and reversible decrease in time spent active from 8.02 ± 2.54% to 0.94 ± 0.46% (*n* = 6, *P* < 0.05; Fig. 8, *A* and *B*). This result therefore supports the conclusion that NO is not only involved in activating locomotion at the level of the brain stem, but that it is also released by descending projections within the spinal cord (see Ramanathan et al. 2006), where it is likely to play a role in sustaining ongoing locomotor activity.

## DISCUSSION

We have explored the role of NO in modulating spinal motor output that drives rhythmic locomotor movements in *Xenopus* tadpoles, and how this role changes during pre-metamorphic development. The larval phase of *Xenopus* development comprises two distinct epochs: in the initial period (stages 37/38 to 43/44; Fig. 1), following release from the egg membrane, the hatchling tadpole normally lies dormant for the first few days while feeding from its yolk sac. Then, once the mouth, gut and anus become functional (from stage 45 onwards), the tadpole enters a more prolonged period of free swimming, filter feeding larval life. During the sessile postembryonic stages of development, tadpoles will swim when stimulated, with swimming generated by a well-characterized CPG circuit located in the spinal cord and caudal hindbrain (Roberts et al. 2010). Endogenous release of the gaseous modulator NO or exogenous application of NO donors at these stages decreases the duration and frequency of sensory-evoked swimming (McLean and Sillar 2000, 2004) and thus reduces locomotor activity in a manner consistent with the animal's mostly sedentary lifestyle (Fig. 4*A*). After the onset of free swimming, tadpoles are continuously active in a cruising/food-seeking mode via caudal tail muscle contractions, with recruitment of more rostral segments occurring during the propulsive accelerations of faster, sprint swimming (Hoff and Wassersug 1986). As we show here, there is a relatively abrupt switch in the effects of NO that coincides with the time point at which the tadpoles enter the free swimming stages of development. From stage 47, NO donors increase the probability of occurrence of fictive swimming episodes and thus promote increased locomotor activity, although without significantly affecting either the duration of individual episodes or the duration and frequency of motor bursts within episodes (Figs. 4*B* and 5 and Table 1). While these donor experiments indicate a complete reversal of NO's central nervous effects around stage 47, there was evidence that endogenous NO is incapable of fully activating the spinal locomotor system until slightly later in development. While removing endogenous NO from the nervous system reduces swimming output from stage 48 (Fig. 6), the same manipulation has a clearly less potent effect at earlier stage 47 (Fig. 4*Biii*). Indeed, it is perhaps predicable that endogenous NO levels will be lower at the younger transitional stages, and, besides, the switch from inhibition to de novo excitation by NO might be expected to traverse a developmental phase when emerging excitatory effects are at least partly competing with decreasing but residual inhibitory influences. The idea of such a transitional period is also evident from our behavioral data (Fig. 1) that show swimming activity is not fully mature until stage 47, despite a clear trend in this direction from as early as stage 45.

The inhibitory effects of NO on swim ED at early stages of *Xenopus* development are proposed to result from the presynaptic facilitation of a descending GABAergic “stopping” pathway, normally activated following contact of the rostral cement gland with an object in the environment (Boothby and Roberts 1992). The signal from cement gland afferent fibers is relayed to the spinal cord by mid-hindbrain reticulospinal neurons (McLean and Sillar 2002). Presumably this enhancing effect of NO on GABAergic inhibitory transmission is reduced or completely absent in older free-swimming larval stages when manipulations of the nitrergic system are without obvious effect on swim ED. This makes behavioral “sense” since the afferent component of the stopping pathway from the cement gland is known to have completely degenerated by stage 45/46 (Boothby and Roberts 1992). In any case, neither the presence of such a pathway nor its facilitation by NO are compatible with a later larval lifestyle phase during which swimming is almost continuous. Furthermore, such a disengagement from tonic inhibitory brain stem inputs to the spinal cord could contribute to the observed increase in the spontaneous expression of swim episodes over this period of development (Fig. 1, *B* and *C*). In parallel, the slowing effect of NO on swim frequency at early postembryonic stages results from its potentiation of glycinergic inhibitory transmission in the spinal cord, a process involving NO metamodulation of noradrenergic transmission in the brain stem, which in turn causes presynaptic facilitation of glycine release (McLean and Sillar 2004). The lack of significant effect of NO on swim frequency at free-swimming stages (for example, see Fig. 5) suggests either a similar disconnection of NO influence from the noradrenergic pathway, and/or that the nitrergic modulation of glycinergic synapses persists but is now somehow overridden by opposing excitatory influences.

Here, we further show that, following the developmental switch in NO's modulatory impact on locomotion from inhibition of swim frequency and swim ED to an enhancement of spontaneous swim episode occurrence, this latter facilitatory effect of NO occurs primarily in the brain stem (Fig. 7). While NO donors applied selectively to the spinal cord reduce swimming activity, the same drugs increase activity when applied either to the entire preparation or more selectively to the brain stem (Fig. 7, *B* and *C*). However, we also found that after activity had been increased by brain stem NO donor exposure, removal of NO from the spinal cord using an NO scavenger has the opposite effect of dramatically decreasing rhythmic locomotor output (Fig. 8). This is an intriguing finding because it implies that brain stem NO simultaneously triggers spinal CPG activity and causes an excitatory effect in the spinal cord, which then appears to play a supporting role in the expression of CPG output. Since NO is widely known to potentiate glutamatergic signaling in other systems (for example, Garthwaite 2008) and since some of the descending projection neurons may be both nitrergic and glutamatergic (Ramanathan et al. 2006), the spinal excitatory effect of NO could be due to a co-transmitter role in the pathway that initiates locomotion. However, the reduction in activity following NO donor applications to the spinal cord alone suggests that there may be an optimal tissue NO concentration required for proper locomotor network operation.

### 

#### Locomotor output modulation by NO and DA via parallel pathways.

As mentioned above, in earlier stages of *Xenopus* development, NO mediates its inhibitory effects primarily by enhancing the release of other inhibitory neurotransmitters. Specifically, these NO-derived effects involve a direct increase in GABA release and an indirect increase in glycine release via a facilitation of noradrenergic signaling (McLean and Sillar 2002, 2004). This is in accordance with a general role for NO in facilitating synaptic transmission (for review, see Garthwaite 2008). Indeed, NO is also known to be involved in governing aminergic signaling (for review, see Kiss 2000), including potentiating striatal DA effects via DA re-uptake blockade (Kiss et al. 1999). Furthermore, a study in larval zebrafish showed that the behavioral increase in spontaneous swimming between 3 and 5 dpf is replicated in vitro and is dependent on dopaminergic signaling (Thirumalai and Cline 2008). Based on such data and the fact that both NO and DA are capable of modulating the spontaneous locomotor activity in very similar ways in pro-metamorphic *Xenopus* tadpoles (this study; c.f., Clemens et al. 2012), we were interested in determining whether there was a direct metamodulatory interaction between the two systems. By applying the D1 receptor antagonist SCH to the spinal cord, we blocked the known pathway for DA's excitatory action on locomotor output in pro-metamorphic tadpoles (Clemens et al. 2012). However, the subsequent increase in fictive locomotion triggered by NO donors to the brain stem strongly suggested that the nitrergic and dopaminergic modulatory systems act in parallel rather than in a serial manner to modulate output of the spinal CPG (Fig. 8). Interestingly, in zebrafish, dopaminergic signaling in the brain but not the spinal cord was found to suppress locomotor activity at 3 dpf (Thirumalai and Cline 2008), whereas between 3 and 4 dpf, DA acting within the spinal cord underlies a maturational change when locomotor activity is dramatically increased (Lambert et al. 2012). This earlier work and our own thus further highlight the importance of both region- and stage-specific application of drugs to the CNS of in vitro experimental preparations.

Finally, one alternative explanation for NO's facilitation of older larval swimming is that noradrenergic neurons of the locus coeruleus are activated in response to elevated brain stem NO levels, which in turn leads to the activation of downstream spinal CPG circuitry. Precedence for this idea exists in the mammalian spinal cord where descending noradrenergic modulation has been found to upregulate recurrent excitation of spinal motorneurons (Machacek and Hochman 2006).

## GRANTS

This study was supported by Projet International de Coopération Scientifique of the French Centre National de la Recherche Scientifique and a LabEx BRAIN Visiting Professorship to K. T. Sillar. S. P. Currie was a Biotechnology and Biological Sciences Research Council research student. N. W. Scott was an MPhil student supported in part by the E & RS Research Fund of the University of St. Andrews.

## DISCLOSURES

No conflicts of interest, financial or otherwise, are declared by the author(s).

## AUTHOR CONTRIBUTIONS

Author contributions: S.P.C., D.C., J.S., and K.T.S. conception and design of research; S.P.C., D.C., N.W.S., and K.T.S. performed experiments; S.P.C., D.C., N.W.S., J.S., and K.T.S. analyzed data; S.P.C., D.C., N.W.S., J.S., and K.T.S. interpreted results of experiments; S.P.C., D.C., N.W.S., and K.T.S. prepared figures; S.P.C. and K.T.S. drafted manuscript; S.P.C., D.C., N.W.S., J.S., and K.T.S. edited and revised manuscript; D.C., N.W.S., J.S., and K.T.S. approved final version of manuscript.

## References

[B1] Boothby KM, Roberts A The stopping response of *Xenopus laevis* embryos: behaviour, development and physiology. J Comp Physiol A 170: 171–180, 1992. 1583603 10.1007/BF00196899

[B2] Clemens S, Belin-Rauscent A, Simmers J, Combes D Opposing modulatory effects of D1- and D2-like receptor activation on a spinal central pattern generator. J Neurophysiol 107: 2250–2259, 2012. 22262823 10.1152/jn.00366.2011

[B3] Combes D, Merrywest SD, Simmers J, Sillar KT Developmental segregation of spinal networks driving axial- and hindlimb-based locomotion in metamorphosing *Xenopus laevis*. J Physiol 559: 17–24, 2004. 15235079 10.1113/jphysiol.2004.069542 PMC1665069

[B4] Dale N, Gilday D Regulation of rhythmic movements by purinergic neurotransmitters in frog embryos. Nature 383: 259–263, 1996. 8805702 10.1038/383259a0

[B5] Ferrero R, Rodríguez-Pascual F, Miras-Portugal MT, Torres M Comparative effects of several nitric oxide donors on intracellular cyclic GMP levels in bovine chromaffin cells: correlation with nitric oxide production. Br J Pharmacol 127: 779–787, 1999. 10401570 10.1038/sj.bjp.0702607 PMC1566069

[B6] Foster JD, Dunford C, Sillar KT, Miles GB Nitric oxide-mediated modulation of the murine locomotor network. J Neurophysiol 111: 659–674, 2014. 24259545 10.1152/jn.00378.2013 PMC3921400

[B7] Garthwaite J Concepts of neural nitric oxide-mediated transmission. Eur J Neurosci 27: 2783–2802, 2008. 18588525 10.1111/j.1460-9568.2008.06285.x PMC2610389

[B8] Hoff K, Wassersug RJ The kinematics of swimming in larvae of the clawed frog, *Xenopus laevis*. J Exp Biol 122: 1–12, 1986.

[B9] Jaffrey SR, Erdjument-Bromage H, Ferris CD, Tempst P, Snyder SH Protein S-nitrosylation: a physiological signal for neuronal nitric oxide. Nat Cell Biol 3: 193–197, 2001. 11175752 10.1038/35055104

[B10] Katz P Intrinsic and extrinsic neuromodulation of motor circuits. Curr Opin Neurobiol 5: 799–808, 1995. 8805409 10.1016/0959-4388(95)80109-x

[B11] Kiss JP Role of nitric oxide in the regulation of monoaminergic neurotransmission. Brain Res Bull 52: 459–466, 2000. 10974484 10.1016/s0361-9230(00)00282-3

[B12] Kiss JP, Hennings EC, Zsilla G, Vizi ES A possible role of nitric oxide in the regulation of dopamine transporter function in the striatum. Neurochem Int 34: 345–350, 1999. 10372921 10.1016/s0197-0186(99)00019-4

[B13] Kyriakatos A and El Manira A. Long-term plasticity of the spinal locomotor circuitry mediated by endocannabinoid and nitric oxide signaling. J Neurosci 27: 12664–12674, 2007. 18003846 10.1523/JNEUROSCI.3174-07.2007 PMC6673314

[B14] Kyriakatos A, Molinari M, Mahmood R, Grillner S, Sillar KT, El Manira A Nitric oxide potentiation of locomotor activity in the spinal cord of the lamprey. J Neurosci 29: 13283–13291, 2009. 19846716 10.1523/JNEUROSCI.3069-09.2009 PMC6665181

[B15] Lambert AM, Bonkowsky JL, Masino MA The conserved dopaminergic diencephalospinal tract mediates vertebrate locomotor development in zebrafish larvae. J Neurosci 32: 13488–13500, 2012. 23015438 10.1523/JNEUROSCI.1638-12.2012 PMC3481997

[B16] Machacek DW, Hochman S Noradrenaline unmasks novel self-reinforcing motor circuits within the mammalian spinal cord. J Neurosci 26: 5920–5928, 2006. 16738234 10.1523/JNEUROSCI.4623-05.2006 PMC2680501

[B17] McLean DL, Sillar KT The distribution of NADPH-diaphorase-labelled interneurons and the role of nitric oxide in the swimming system of *Xenopus laevis* larvae. J Exp Biol 203: 705–713, 2000. 10648212 10.1242/jeb.203.4.705

[B18] McLean DL, Sillar KT Spatiotemporal pattern of nicotinamide adenine dinucleotide phosphate-diaphorase reactivity in the developing central nervous system of premetamorphic *Xenopus laevis* tadpoles. J Comp Neurol 437: 350–362, 2001. 11494261 10.1002/cne.1288

[B19] McLean D, Sillar KT Nitric oxide selectively tunes inhibitory synapses to modulate vertebrate locomotion. J Neurosci 22: 4175–4184, 2002. 12019335 10.1523/JNEUROSCI.22-10-04175.2002 PMC6757640

[B20] McLean D, Sillar KT Metamodulation of a spinal locomotor network by nitric oxide. J Neurosci 24: 9561–9571, 2004. 15509743 10.1523/JNEUROSCI.1817-04.2004 PMC6730165

[B21] Miles GB, Sillar KT Neuromodulation of vertebrate locomotor control networks. Physiology (Bethesda) 26: 393–411, 2011. 22170958 10.1152/physiol.00013.2011

[B22] Muntz L Myogenesisin the trunk and leg during development of the tadpole of *Xenopus laevis* (Daudin 1802). J Embryol Exp Morphol 33: 757–774, 1975. 1176869

[B23] Nieuwkoop PD, Faber J Normal Tables of Xenopus laevis (Daudin). Amsterdam: North Holland, 1956.

[B24] Ramanathan S, Combes D, Molinari M, Simmers J, Sillar KT Developmental and regional expression of NADPH-diaphorase/nitric oxide synthase in spinal cord neurons correlates with the emergence of limb motor networks in metamorphosing *Xenopus laevis*. Eur J Neurosci 24: 1907–1922, 2006. 17067294 10.1111/j.1460-9568.2006.05057.x

[B25] Reith CA, Sillar KT Pre- and postsynaptic modulation of spinal GABAergic neurotransmission by the neurosteroid, 5-pregnan-3-ol-20-one. Brain Res 770: 202–212, 1997. 9372220 10.1016/s0006-8993(97)00809-3

[B26] Roberts A, Li WC, Soffe SR How neurons generate behavior in a hatchling amphibian tadpole: an outline. Front Behav Neurosci 4: 16, 2010. 20631854 10.3389/fnbeh.2010.00016 PMC2903309

[B27] Song J, Kyriakatos A, El Manira A Gating the polarity of endocannabinoid-mediated synaptic plasticity by nitric oxide in the spinal locomotor network. J Neurosci 32: 5097–5105, 2012. 22496555 10.1523/JNEUROSCI.5850-11.2012 PMC6622082

[B28] Thirumalai V, Cline HT Endogenous dopamine suppresses initiation of swimming in prefeeding zebrafish larvae. J Neurophysiol 100: 1635–1648, 2008. 18562547 10.1152/jn.90568.2008 PMC2544474

[B29] van Mier P, Armstrong J, Roberts A Development of early swimming in *Xenopus laevis* embryos: myotomal musculature, its inervation and activation. Neuroscience 32: 113–126, 1989. 2586744 10.1016/0306-4522(89)90111-5

